# Intrinsic Calcification of the Permanent Tooth-Pulp

**Published:** 1856-06

**Authors:** 


### INTRINSIC CALCIFICATION OF THE PERMANENT TOOTH-PULP.

The May number of the Dental Recorder, continues the publication of an article on the *intrinsic* calcification of the permanent tooth pulp, by S. James A. Salter, M. D., &c.

This is a valuable paper, and we should like to have given our readers more copious extracts from it. In relation to the changes which occur in the production of normal dentine, and some of the pathological conditions producing osteo-dentine, &c., he remarks that:

It will be necessary, before entering upon the details of this subject to make a few remarks upon the changes which occur in the production of normal dentine, and the manner in which the pulp is reduced to its adult condition, as well as the anatomy of the latter organ; and this will be necessary both as a basis for the explanation of the pathological changes, and also to show the analogies, and at the same time the differences, between the normal process and the pathological changes, which I here endeavor to elucidate.

The formative tooth-pulp is a papilla, consisting of numerous blood-vessels and nerves, distributed through multitudes of cells and nuclei, and an immature connective tissue, and covered in by a basement membrane—the whole constituting a form precisely similar to the dentine of the crown of the future tooth. A series of columnar cells, very similar in appearance to those of columnar epthelium, is found arranged in an even layer upon the surface, covered in only by the basement membrane; from the distal extremities of these cells appear outgrowths, in the form of capillary tubes, and continuous with the cell wall, and these increase in length by the backward recession of the body of the cells, the tubes being thus prolonged inwards: these constitute the animal basis of the dentinal tubes. The intertubular tissue appears to be formed of a hyaline animal structure, in which no histological elements are indicated.

It must be recollected that this process is entirely superficial; that it occurs from without inwards; that no other histological elements enter into the formation of the dentine besides the cells described; and that the nerves and blood-vessels recede as the dentine advances. The "*conversion*" theory, which involved the idea of a complete change of the entire pulp-structures into *normal* dentine, is now known to be altogether erroneous; and this it is especially important to remember in relation to the present subject.

The mode in which the animal material is impregnated with the calcareous in primary dentine is very remarkable, and has a certain similarity to the morbid condition I am about to describe. It has been shown by Czermak, and confirmed and still further elucidated by myself, that after the animal material of dentine has sketched out its anatomical form, it becomes calcified, not by an even gradual impregnation throughout the whole, nor in any relation to the course of its structure, but in isolated globular patches, by the enlargement and fusion of which the whole is formed into an even coherent mass. And I would further remark—what is of value and interest in reference to the present matter—that the animal material is palpably altered at the time of calcification in physical and chemical characters: it is harder, denser, and contains less water in proportion to the animal solid matter.

I have already alluded to osteodentine as the issue under certain circumstances of the morbid change now under consideration, and I would here observe that osteodentine constitutes one of those peculiar forms of dentine called "secondary" on acount of their being after-formations—developed, that is, after the primary system of dentine has been matured. I must add, that the other two forms of secondary dentine, "dentine of repair" (formed by laminæ on the inner surface of the pulp cavity, as repair and recompense for external wear, decay, or fracture) and "dentine-excrescence" (a modular growth of dentine arising spontaneously on the interior of the pulp-cavity, and without disease or apparent cause)—that these forms are in all essential particulars the same as primary dentine, formed and calcified in the same way, having contour lines parallel to the surface, and not involving blood-vessels or nerves.

